# Steroid-free medium discloses oestrogenic effects of the bisphosphonate clodronate on breast cancer cells

**DOI:** 10.1038/sj.bjc.6602181

**Published:** 2004-10-12

**Authors:** F Journe, C Chaboteaux, J-C Dumon, G Leclercq, G Laurent, J-J Body

**Affiliations:** 1 Laboratory of Endocrinology and Bone Diseases, Institut Jules Bordet, Centre des Tumeurs de l’Université Libre de Bruxelles, Brussels, Belgium; 2 Laboratory of Breast Cancer Research, Institut Jules Bordet, Centre des Tumeurs de l’Université Libre de Bruxelles, Brussels, Belgium; 3 Laboratory of Histology, Faculty of Medicine and Pharmacy, Université de Mons-Hainaut, Mons, Belgium

**Keywords:** clodronate, bisphosphonate, breast cancer cells, oestrogen receptor, antioestrogen

## Abstract

Tamoxifen is the standard first-line endocrine therapy for breast cancer, but recent data indicate that it is likely to be replaced by the effective aromatase inhibitors (AIs), in both the metastatic and adjuvant settings. Aromatase inhibitors induce complete oestrogen deprivation that leads to clinically significant bone loss. Several ongoing or planned trials combine AIs with bisphosphonates, even more so that recent data reveal that clodronate may reduce the incidence of bone metastases and prolong survival in the adjuvant setting. Bisphosphonates can inhibit breast cancer cell growth *in vitro*, but they have never been studied in steroid-free medium (SFM), an *in vitro* environment that mimics the effects of AIs *in vivo*. Quite surprisingly, in SFM, clodronate stimulated MCF-7 cell growth in a time- and dose-dependent manner by up to two-fold (crystal violet staining assay), whereas it had no mitogenic activity in complete medium. The bisphosphonate similarly increased the proliferation of IBEP-2 cells, which also express a functional oestrogen receptor (ER), while it weakly inhibited the growth of the ER-negative MDA-MB-231 cells. Expectedly, 17*β*-oestradiol stimulated the growth of MCF-7 and IBEP-2 cells cultured in SFM, and had no effect on MDA-MB-231 cells. Moreover, partial (4-OH-tamoxifen) and pure antioestrogens (fulvestrant, ICI 182,780), in combination with clodronate, completely suppressed the mitogenic effect of the bisphosphonate, suggesting that it was mediated by an activation of ER. In accordance with this view, clodronate induced ER downregulation, weakly increased progesterone receptor expression, and stimulated the transcription of an oestrogen-responsive reporter gene. In conclusion, we report a previously unknown stimulatory effect of clodronate on MCF-7 cells grown in SFM, *in vitro* conditions that are potentially relevant to the use of AIs for breast cancer. Moreover, our data suggest that ER is involved in these effects of clodronate on cancer cell growth.

Breast cancer is the most prominent malignant neoplasm in women, at least in economically developed Western countries. Tamoxifen stands as the standard first-line endocrine therapy for female patients with breast carcinoma expressing oestrogen receptor alpha (ER), who represent the majority of breast cancer cases. Tamoxifen inhibits the growth of breast tumors by competitively antagonising oestrogen effect on cognate receptors (Bardon *et al*, 1984). It also has partial agonist (i.e. oestrogenic) activity which can be beneficial since it prevents bone loss in postmenopausal women (Love *et al*, 1994; Powles *et al*, 1996).

A novel approach for the hormonal therapy of breast carcinoma relies on the use of aromatase inhibitors (AIs) in place of tamoxifen. AIs markedly suppress plasma oestrogen level in postmenopausal women by inhibiting or inactivating aromatase enzymes responsible for the synthesis of oestrogen from androgenic substrates (Brodie and Njar, 2000). They also markedly inhibit intratumoral aromatase activity present in the majority of breast cancers (Harper-Wynne and Dowsett, 2001; Miller *et al*, 2001).

Last generation nonsteroidal AIs, such as letrozole (Femara®) and anastrozole (Arimidex®), are widely recommended as second-line hormonal agents for postmenopausal breast cancer patients (Goss and Strasser, 2001) and they have recently proven to be superior to tamoxifen as first-line treatment for advanced disease (Bonneterre *et al*, 2000; Nabholtz *et al*, 2000; Mouridsen *et al*, 2001) and, even more importantly, in the adjuvant setting as well (Baum *et al*, 2002). Thus, AIs are bound to progressively replace tamoxifen for the first-line treatment of postmenopausal patients with ER-positive cancers (Smith and Dowsett, 2003).

Nevertheless, the risk of osteoporotic fractures is likely to increase with the use of AIs since the maintenance of bone density partly depends on oestrogen, even in postmenopausal women. On the other hand, tamoxifen reduces bone mineral loss through its weak agonist effect, at least in postmenopausal women (Powles *et al*, 1996). Short-term use of letrozole has been shown to be associated with an increase in bone resorption markers in plasma and urine (Harper-Wynne *et al*, 2002; Heshmati *et al*, 2002) and adjuvant therapy with anastrozole is associated with a higher incidence of fractures than adjuvant therapy with tamoxifen (Boccardo *et al*, 2001; Baum *et al*, 2002).

It should be possible to prevent bone demineralization associated with oestrogen deprivation therapy by combining the latter with the use of bisphosphonates (Cummings *et al*, 1998). Bisphosphonates are indeed potent inhibitors of osteoclasts and represent the reference therapy for the management of postmenopausal osteoporosis (Fleisch, 2003). Moreover, many studies indicate that they prevent the development of metastatic bone diseases in animals (Yoneda *et al*, 2000). A few human trials have already been performed to evaluate oral clodronate in this setting. Although the clinical usefulness of this compound in the adjuvant treatment of breast cancer remains somewhat controversial (Diel *et al*, 1998; Saarto *et al*, 2001; Coleman, 2003), the largest trial so far has shown that clodronate can decrease the incidence of bone metastasis and prolong survival (Powles *et al*, 2002). Several trials are currently in progress or planned to disclose possible combined effect of AIs and various bisphosphonates in the treatment of breast cancers, with the prospect of preventing bone mineral loss and reducing the incidence of bone metastasis.

Bisphosphonates are also able to inhibit breast cancer cell growth *in vitro* (for a review, see Neville-Webbe *et al*, 2002). However, all studies reported so far were performed in steroid-containing medium. The present work has investigated the effects of clodronate on the growth of the ER-positive MCF-7 breast cancer cell line cultured in steroid-free medium (SFM) in order to mimic the effect of AIs *in vivo*. We have also analysed the effect of clodronate in combination with ER agonist and antagonists, with particular emphasis on ER expression and activity.

## MATERIAL AND METHODS

### Cell culture conditions

The ER-positive MCF-7 breast cancer cell line (ATCC HTB-22) was initially obtained in 1977 from the Michigan Cancer Foundation (Detroit, MI, USA). IBEP-2 cell line was previously established in our laboratory from a pleural effusion due to metastatic breast carcinoma (Siwek *et al*, 1998) and also expresses functional ER. MDA-MB-231 breast carcinoma cells (ATCC HTB-26) lack ER expression.

All plastic flasks, dishes and multiwell plates for cell culture were obtained from Nunc™ (Naperville, IL, USA). Cells were cultured at 37°C in a humidified 95% air and 5% CO_2_ atmosphere.

For routine maintenance, cells were cultured in 75-cm^2^ flasks containing Eagle's minimum essential medium (MEM) with Phenol Red, supplemented with 10% heat-inactivated fetal calf serum (FCS), and containing standard concentrations of L-glutamine, penicillin and streptomycin (Gibco BRL, Life Technologies, Merelbeke, Belgium). Cells were harvested by trypsinisation (0.1% trypsin – 0.02% EDTA) and subcultured twice a week.

For all experiments, cells were plated in SFM (day 0) consisting of RPMI Medium 1640 without Phenol Red (Gibco BRL, Life Technologies, Merelbeke, Belgium), supplemented with 10% charcoal-stripped FCS as previously described (Devleeschouwer *et al*, 1987). At day 1, the seeding medium was replaced by fresh SFM containing clodronate (gift from Roche Diagnostics GmbH, Mannheim, Germany), 17*β*-oestradiol (E_2_; Sigma, St Louis, MO, USA), 4-hydroxy-tamoxifen (4-OH-TAM; Sigma, St Louis, MO, USA) fulvestrant (ICI 182,780; Tocris, Bristol, UK) at concentrations specified in ‘Results’. In control cultures, drug solution was replaced by an equivalent amount of vehicle. Treatment lasted from 1 to 3 days.

MVLN cells (generously provided by Dr M Pons, INSERM U58, Monpellier, France) are MCF-7 cells stably transfected with the oestrogen-response element cloned upstream the luciferase reporter gene (Demirpence *et al*, 1993). They were cultured in the same conditions as that used for MCF-7 cells.

### Crystal violet staining

Cell number was assessed indirectly by staining with crystal violet dye, since the total amount of cell-bound dye is proportional to cell number (Lee *et al*, 2001). Cancer cells were seeded in 96-well plates (density 5000 cells well^−1^) in SFM or complete medium, and cultured for 24 h. Cells were then exposed to clodronate, E_2_, antioestrogens or vehicle as described in ‘Results’. After incubation, the medium was removed and cells were gently rinsed with PBS. Cells were fixed with 1% glutaraldehyde/PBS for 15 min and stained with crystal violet (0.1% w/v in dd H_2_O). Cell surfaces were destained under slightly floating tap water for 15 min and subsequently lysed with 0.2% Triton X-100 (v/v in dd H_2_O). The absorbance was measured at 550 nm using a Microplate Autoreader EL309 (BIO-TEK Instruments). Blanks (wells containing medium alone) were processed in parallel, and their values were subtracted from those of the sample plates.

### Western blot analysis (ER and PgR)

Oestrogen receptor (ER) and progesterone receptor (PgR) amounts per milligram proteins were determined by Western blotting. Cells were plated in 60-cm^2^ Petri dishes (density 10 000 cells cm^−2^) in SFM and cultured for 24 h. After incubation with clodronate, E_2_ or vehicle as specified in ‘Results’, cell cultures were rinsed twice with tris-buffered saline (TBS, 50 mM Tris-HCl pH 7.5 and 150 mM NaCl) and harvested using 500 *μ*l lysis buffer (TBS, 0.5% sodium deoxycholate, 1% Nonidet P-40, 0.1% SDS, 50 mM NaF, 0.1 mM Na_3_VO_4_ and 5 mM EDTA) with freshly added proteolysis inhibitors. Total cell lysates were mixed with standard electrophoresis sample buffer. Denatured samples (20 and 40 *μ*g of cellular proteins for ER and PgR assays, respectively) were subjected to SDS–PAGE under reducing conditions. Proteins were subsequently electrotransferred onto a nitrocellulose membrane (Amersham Pharmacia Biotech, Roosendaal, The Netherlands). The membrane was then incubated for 3 h at room temperature in a blot solution (10 mM Tris-HCl pH 8, 150 mM NaCl, 0.05% Tween 20 and 7% skimmed milk) to block nonspecific binding, then incubated overnight at 4°C in a fresh blot solution containing polyclonal rabbit anti-human ER*α* antibody (HC-20, Santa Cruz Biotechnology, Santa Cruz, CA, USA) diluted 1 : 5000 or monoclonal mouse anti-human PgR (A/B isoforms) antibody (NCL-PGR-AB, Novocastra Laboratories, Newcastle upon Tyne, UK) diluted 1 : 500. Immunoblots were then incubated for 2 h at room temperature in a blot solution containing peroxidase-labelled donkey anti-rabbit IgG antibody (1 : 5000) or peroxidase-labelled sheep anti-mouse IgG antibody (1 : 5000) (Amersham Pharmacia Biotech, Roosendaal, The Netherlands), depending on primary used antibody. The bound peroxidase activity was revealed using the Lumi-Light Western Blotting Substrate (Roche Diagnostics GmbH, Mannheim, Germany). The immunoreactive band intensity was estimated using a computer-assisted gel scanning densitometer (GS-710 Callibrated Imaging Densitometer) and Quantity One software, both from Bio-Rad (Hercules, CA, USA).

### Luciferase induction assay

MVLN cells were used to study the transcriptional activity of ER by determining ER-induced luciferase activity (Demirpence *et al*, 1993) using the Luciferase Assay System from Promega (Madison, WI, USA). Cells were plated in six-well plates (density 100 000 cells well^−1^) in SFM and were cultured for 72 h. At the end of treatment with clodronate, E_2_, antioestrogens or vehicle as described in ‘Results’, the medium was removed and the cell monolayers rinsed twice with PBS. A minimal volume (250 *μ*l) of a five-fold diluted lysis solution (Promega E153A) was added and the culture was maintained under mild agitation for 20 min to extract luciferase. Detergent-lysed cells were scraped and suspensions were clarified by centrifugation for 5 min at 10 000 **g**. Finally, 20 *μ*l of extracts were mixed at room temperature with 100 *μ*l of a luciferase reactant medium (Promega E151A/E152A) prepared according to the manufacturer's protocol. Luminescence was measured in a Berthold luminometer (Lumat LB 9507). Luciferase induction was expressed in arbitary units (relative luciferase units, RLU) calculated per mg of protein and data are given in percent of mean value obtained from untreated cells.

### Protein determination

Protein concentrations in total cell lysates obtained by detergent extraction were determined by the BCA Protein Assay (Pierce, Rockford, IL, USA) using bovine serum albumin (BSA) as standard.

### Statistical analysis

Data are reported as means±s.d. Statistical analysis was performed using analysis of variance (ANOVA) with a statistical significance level arbitrarily set at 0.05 (Fisher's PLSD) (StatView version 4.02).

## RESULTS

### Clodronate-induced stimulation of MCF-7 cell growth in SFM

**Figure 1:**
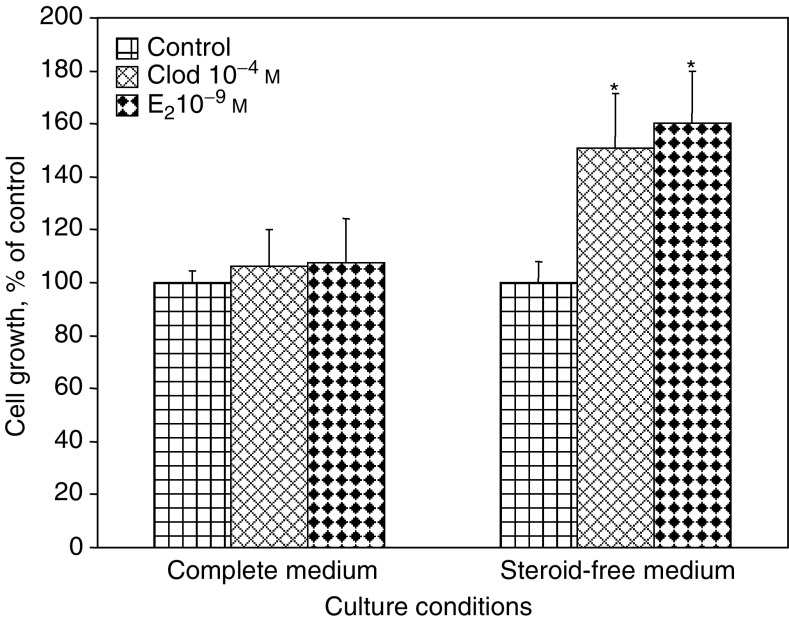
Clodronate stimulated MCF-7 cell proliferation in SFM but not in complete medium. Cancer cells were incubated for 72 h with 10^−4^ M clodronate (Clod), 10^−9^ M E_2_ or vehicle (control) in complete medium or SFM. Cell proliferation was determined by crystal violet staining assay (optical density for control value was 2.129 in complete medium, and 1.197 in SFM). Data are presented as percentages of control values (mean±s.d.). Mean of results is pooled from four experiments (*n*=24). ^*^ANOVA, *P*<0.05 *vs* control.

MCF-7 cells were cultured in SFM for 24 h before additional exposure to 10^−4^ M clodronate or 10^−9^ M 17*β*-oestradiol (E_2_) during 72 h in order to assess cell growth. In these steroid-free culture conditions, clodronate stimulated cell proliferation as evidenced by a marked increase in cell number by 51±21% (mean±s.d., *P*<0.05) compared with controls (Figure 1

). As expected, E_2_ also exerted a growth stimulatory effect (60±20%, *P*<0.05) under similar conditions. By contrast, neither clodronate nor E_2_ stimulated MCF-7 cell growth in medium supplemented with normal, steroid-containing serum (complete medium) (Figure 1). These results suggest that clodronate exerts a mitogenic activity on ER-positive breast cancer cells when the latter are cultured in absence of oestrogens.

**Figure 2:**
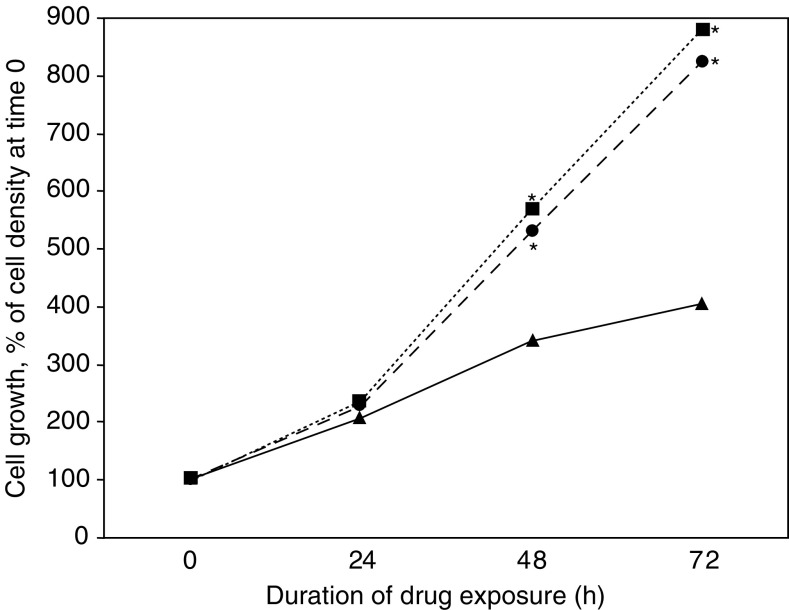
Time-course experiments. Cells were cultured for up to 3 days in SFM containing 10^−4^ M clodronate (circle), 10^−9^ M E_2_ (square) or vehicle (control, triangle). Measurement was performed as described in Figure 1. ^*^ANOVA, *P*<0.05 *vs* control.

Time-course experiments performed over 72 h indicated that clodronate increased cell proliferation in a time-dependent manner similar to what is seen with E_2_ (Figure 2

). Enhancement of cell growth was significant (*P*<0.05) after 48 h of exposure to either compound.

**Figure 3:**
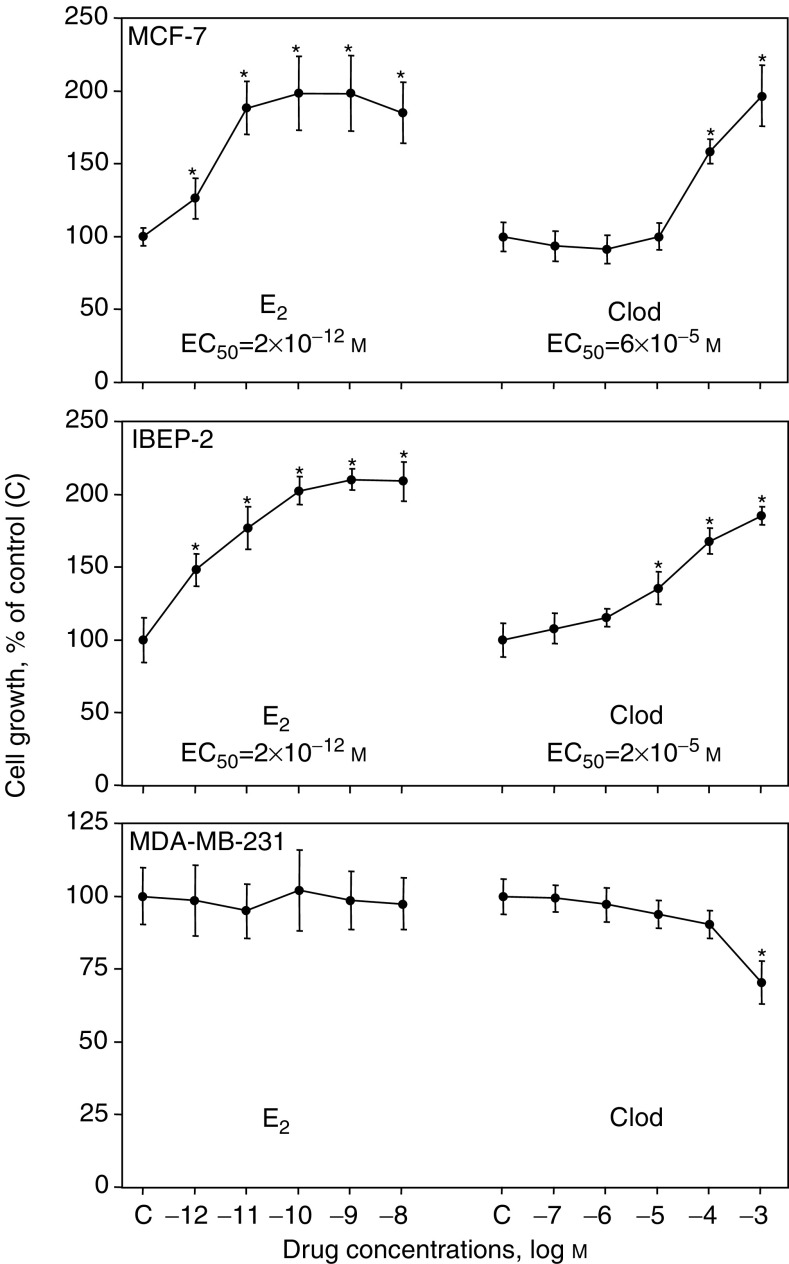
Dose-dependent effects of 17*β*-oestradiol and clodronate on the proliferation of MCF-7, IBEP-2 and MDA-MB-231 cells. Breast cancer cells were treated for 72 h with increasing concentrations of E_2_ (10^−12^–10^−8^ M), clodronate (Clod) (10^−7^–10^−3^ M) or vehicle (control) in SFM. The rest is the same as in Figure 1. ^*^ANOVA, *P*<0.05 *vs* control.

The dose–response relationships characterising the mitogenic effect of E_2_ or clodronate on hormone-deprived MCF-7 cells after 72 h of culture are illustrated in Figure 3

(upper panel). Clodronate (10^−7^–10^−3^ M) induced a dose-dependent response with a maximal stimulation of about two-fold at 10^−3^ M. 17*β*-oestradiol tested at concentrations ranging from 10^−12^ to 10^−8^ M produced a typical sigmoid dose–response curve with a maximal response similar to the one observed for clodronate but occurring at a much lower concentrations. The EC_50_ values (concentrations leading to half maximal stimulation) were around 6 × 10^−5^ and 2 × 10^−12^ M for clodronate and E_2_, respectively. Of note, because of the higher doses that were required, no true plateau phase was reached for clodronate. However, by analogy with the dose–response curve obtained with E_2_, the two-fold stimulation of growth induced by 10^−3^ M clodronate in MCF-7 cells was considered as the maximum effect.

Moreover, we obtained closely similar findings using IBEP-2 cell line, which has recently been reported as an oestrogen-responsive cell line closely resembling the MCF-7 cell line (Journe *et al*, 2004a). We indeed showed that clodronate as well as E_2_ stimulated the proliferation of IBEP-2 cells cultured in SFM (Figure 3, middle panel), indicating that the mitogenic stimulation induced by the bisphosphonate was not restricted to the MCF-7 cells. By contrast, clodronate weakly inhibited the proliferation of MDA-MB-231 cells which are ER-negative breast cancer cells, thus nonsensitive to E_2_ stimulation (Figure 3, bottom panel). Altogether, these data suggest that ER status may be important in the mitogenic effects of clodronate.

### Suppression of clodronate-induced MCF-7 cells growth by antioestrogens

**Figure 4:**
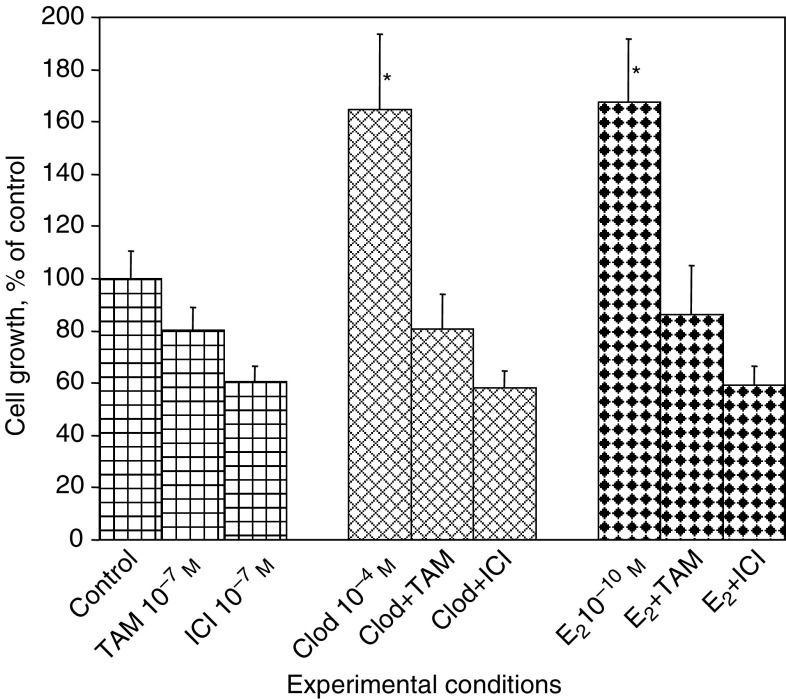
Clodronate-induced cell growth was suppressed by antioestrogens. MCF-7 cells were exposed for 3 days to 10^−4^ M clodronate (Clod), 10^−10^ M E_2_, 10^−7^ M 4-OH-TAM (TAM), 10^−7^ M fulvestrant (ICI) or vehicle (control) in SFM. The rest is the same as in Figure 1. ^*^ANOVA, *P*<0.05 *vs* control.

As suggested above (see Figures 1 and 3), the ER pathway was somehow involved in the mitogenic effect of clodronate. We tested this hypothesis by using two ER antagonists: the partial antioestrogen 4-OH-TAM and the pure antioestrogen fulvestrant (ICI 182,780). IN SFM, exposure of MCF-7 cells to 10^−7^ M antioestrogen for 72 h reduced cell populations by 20±9% (mean±s.d., *P*<0.05) (4-OH-TAM) and by 40±6% (*P*<0.05) (fulvestrant) (Figure 4

). This ability of antioestrogens to impede the growth of ER-positive breast cancer cells (either by interfering with cell proliferation and/or by inducing cell apoptosis) even in the absence of oestrogenic stimulation has also been found by other investigators (Jensen *et al*, 2003). Simultaneous addition of partial or pure antioestrogen with clodronate completely abolished the mitogenic effects of the bisphosphonate and decreased cell growth to values similar to those observed in the presence of antioestrogen alone. As expected, the proliferative response to 10^−10^ M E_2_ was similarly suppressed by concomitant addition of 4-OH-TAM or fulvestrant (Figure 4). Interestingly, previous exposure of MCF-7 cells to antioestrogen for 8 h prior to treatment with clodronate was as efficient as the addition of antioestrogen in combination with clodronate in suppressing the mitogenic effect of the latter (data not shown). These data suggest that preliminary blockade of ER-mediated signalling was sufficient to suppress clodronate-induced cell proliferation, and indicate that clodronate may indeed act through the ER pathway.

### Oestrogenic effects of clodronate in SFM: downregulation of oestrogen receptor, induction of progesterone receptor and transactivation of an oestrogen-responsive reporter gene

As suggested by the use of antioestrogens (see Figure 4), the mitogenic action of clodronate might be mediated by ER. This led us to investigate ER expression and activity under clodronate exposure.

**Figure 5:**
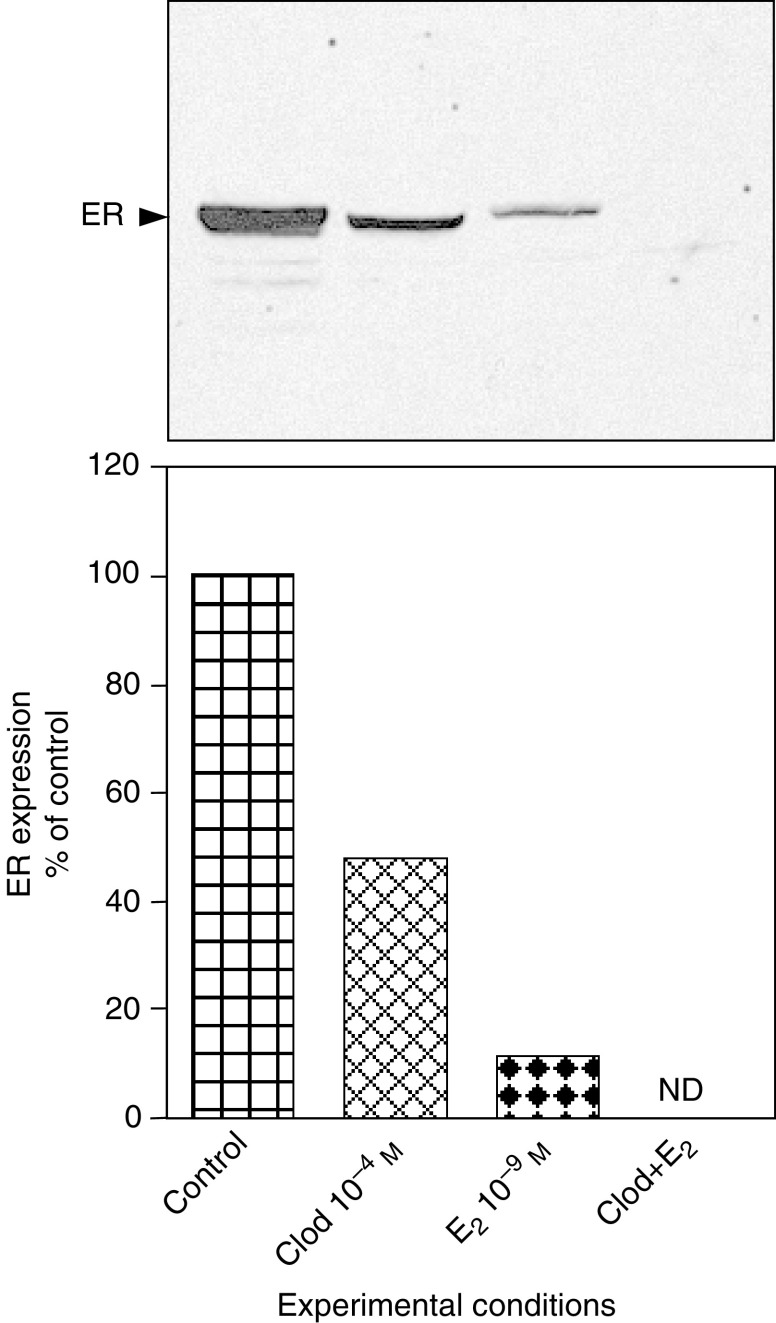
Clodronate downregulated oestrogen receptor expression as assessed by Western blot. MCF-7 cells were incubated for 24 h with 10^−4^ M clodronate (Clod), 10^−9^ M E_2_, Clod+E_2_ or vehicle (control) in SFM. Equal quantities of proteins (20 *μ*g) were subjected to 10% SDS–PAGE and electrotransferred onto nitrocellulose membranes. Immunodetection was performed with anti-human ER antibody raised against its F domain. Blots show representative experiments performed twice. Quantitative data were obtained from densitometric analyses and are presented as percentages of control values (mean). ND=not detectable.

We determined the effect of clodronate on ER expression by Western blot analysis using an antibody raised against the F domain of the receptor (Figure 5

). The amount of immunoreactive ER was determined in MCF-7 cells after 24 h of treatment with clodronate and/or E_2_ in SFM. Densitometric analysis allowed semiquantitative evaluation of ER protein changes. We found that 10^−4^ M clodronate decreased ER amount by about 50%. As expected, 10^−9^ M E_2_ led to a more drastic downregulation of the ER protein. Interestingly, the combination of clodronate and E_2_ induced a complete disappearance of the receptor, suggesting that both compounds may produce additive effects on ER downregulation (Figure 5), and that the pathway for ER downregulation differs according to the drug.

**Figure 6:**
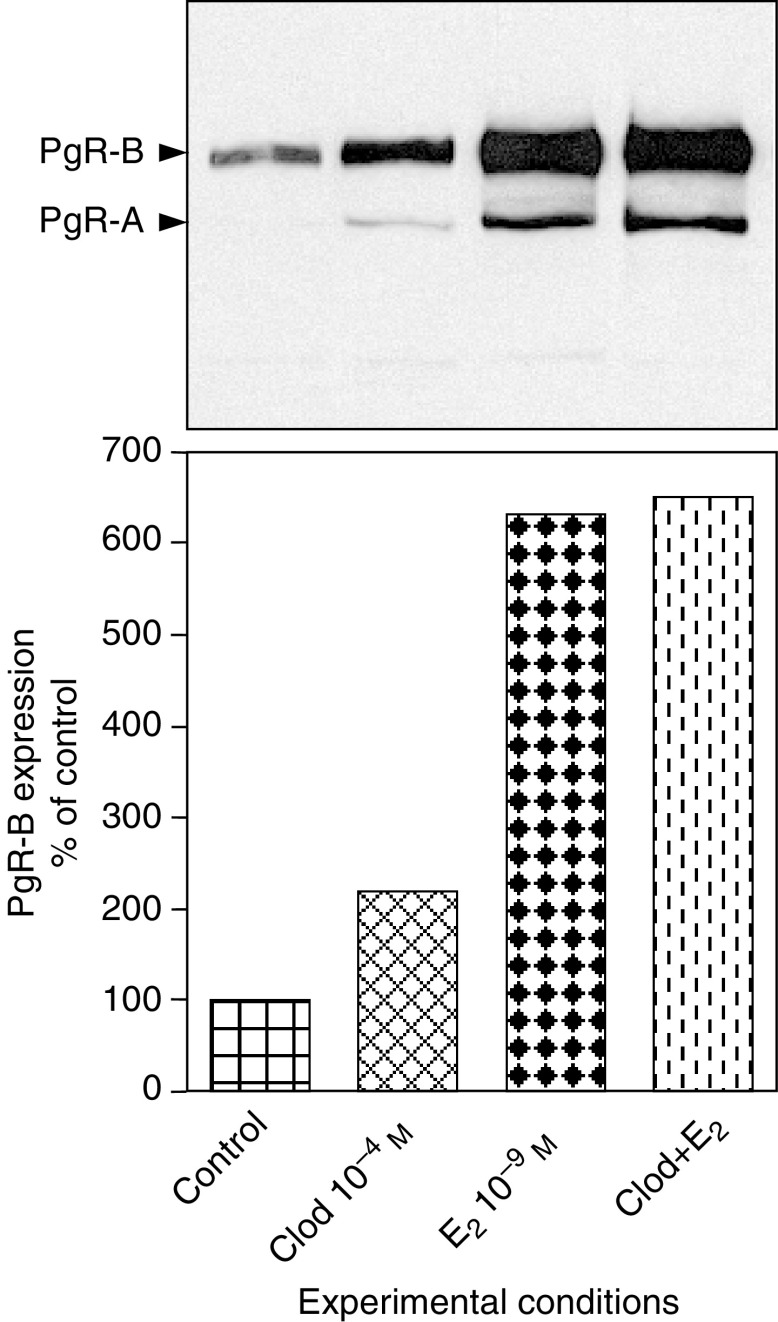
Clodronate induced progesterone receptor expression as determined using Western blot. MCF-7 cells were incubated for 72 h in SFM containing 10^−4^ M clodronate (Clod), 10^−9^ M E_2_, Clod+E_2_ or vehicle (control). Equal amounts of proteins (40 *μ*g) were subjected to 8% SDS–PAGE and electrotransferred onto nitrocellulose membranes. Immunodetection was assessed with anti-human PgR antibody raised against A/B isoforms of the receptor. Blots show representative experiments performed twice. Quantitative data were obtained from densitometric analyses of the B-isoform and are presented as percentages of control values (mean).

We then investigated if clodronate could induce ER-mediated gene transactivation. The effect of clodronate on the oestrogen-inducible PgR gene was assessed by Western blot analysis using an antibody raised against the A/B isoforms of human PgR. Exposure of MCF-7 cells for 72 h to 10^−4^ M clodronate in SFM doubled the expression of PgR-B isoform (114 kDa, the isoform known to function as a transcriptional activator) (Figure 6

). This induction was much less than the one observed with 10^−9^ M E_2_, which increased the PgR level by more than six-fold. The combination of clodronate and E_2_ did not further increase the amount of PgR (possible upper limit with this method beyond which no further increase in protein level can be documented). In control conditions, the A isoform of PgR (94 kDa, the isoform known to act as a transcriptional inhibitor) was not detectable in MCF-7 cells. Small amounts of the A isoform were observed after clodronate stimulation. Here also, E_2_ exerted a more pronounced effect on PgR-A expression. The B/A ratios determined for clodronate- and E_2_-induced stimulation were, however, similar (averaging 15), suggesting no difference in ER-mediated gene transactivation.

**Figure 7:**
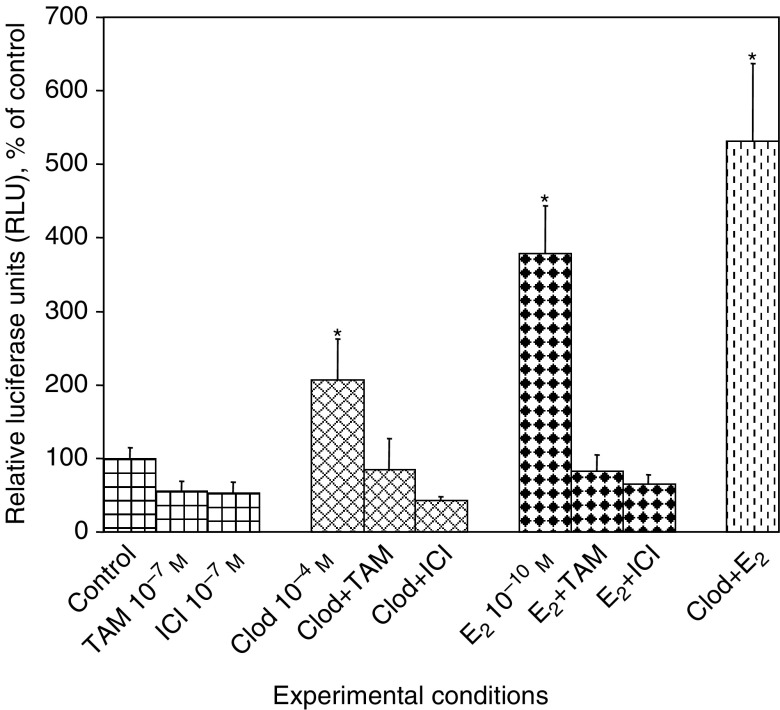
Clodronate stimulated ER transcriptional activity. MVLN cells were incubated in SFM for 24 h with 10^−4^ M clodronate (Clod), 10^−10^ M E_2_, 10^−7^ M 4-OH-TAM (TAM), 10^−7^ M fulvestrant (ICI), combination of Clod or E_2_ with antioestrogens, Clod+E_2_ or vehicle (control) in SFM. Luciferase activities were normalised with protein levels. Data are given as percentages of control values (mean±s.d.). Experiments were performed four times in replicate (*n*=8). ^*^ANOVA, *P*<0.05 *vs* control.

Finally, the transcriptional activity of ER was investigated in MVLN cells obtained by stable transfection of MCF-7 cells with an oestrogen-responsive luciferase reporter gene. Culture for 24 h in SFM containing 10^−4^ M clodronate resulted in a significant increase in ER-induced luciferase expression by 106±56% (mean±s.d., *P*<0.05) (Figure 7

). Reporter gene transactivation caused by 10^−9^ M E_2_ was stronger (279±64%, *P*<0.05). The activation of the reporter gene was further increased when clodronate and E_2_ were combined (431±106%, *P*<0.05). The stimulation of luciferase activity by clodronate and E_2_ were both completely abrogated by simultaneous incubation with 4-OH-TAM or fulvestrant, again suggesting that Clod may specifically induce ER activation.

## DISCUSSION

Bisphosphonates, including clodronate, are widely used for the treatment of metastatic bone disease (Body *et al*, 1998; Lipton, 2003). In particular, they are administered to alleviate skeletal complications resulting from osteoclast-mediated tumour bone disease associated with breast and prostate cancers, and multiple myeloma. Although human data are still scarce, a double-blind trial involving more than 1000 breast cancer cases after surgery indicates that a 2-year treatment with clodronate can reduce the incidence of bone metastases by about one-half, and prolong patient survival (Powles *et al*, 2002).

Bisphosphonates are also currently evaluated in combination with third-generation AIs (Coleman, 2003), which appear to be more efficient than tamoxifen as a first-line treatment of advanced breast cancer (Bonneterre *et al*, 2000; Nabholtz *et al*, 2000; Mouridsen *et al*, 2001), as well as in the adjuvant setting (Baum *et al*, 2002). Thus, the administration of bisphosphonates, notably clodronate, in combination with AIs seems to be an attractive choice in order to prevent the risk of bone loss associated with complete oestrogen deprivation therapy, with the additional possibility of simultaneously decreasing the incidence of bone metastases (Cummings *et al*, 1998).

In our study, we investigated the effects of clodronate on the proliferation of MCF-7 cells cultured in SFM, an *in vitro* model that mimics breast tumours exposed to treatment combining the bisphosphonate clodronate and AI-induced oestrogen deprivation. Quite surprisingly, we found that clodronate stimulated the proliferation of MCF-7 cells in these particular conditions. Moreover, in the same conditions, clodronate exhibited oestrogen-like activity, inducing the downregulation of ER, the expression of progesterone receptor and the transactivation of a reporter gene. Hence, clodronate might increase MCF-7 cell proliferation through specific ER activation, as suggested by a complete suppression of its mitogenic effects by partial or pure antioestrogens.

The concentration of clodronate (10^−4^ M) that was needed to stimulate the growth of MCF-7 cells in SFM appears to be at least 10-fold higher than the circulatory levels in patients treated with bisphosphonates (Osterman and Lauren, 1991; Villikka *et al*, 2002). Nevertheless, it has been shown that bisphosphonates accumulate in bone (Lin *et al*, 1992), and that their effective local concentrations at sites of active bone resorption are much higher than serum levels, and may reach up to 10^−3^ M in the resorption lacunae (Sato *et al*, 1991). It is unclear whether the bioavailability of bone bisphosphonates for cancer cells would result in effective concentration as high as 10^−4^ M. However, we can speculate that the close association of breast cancer cells in chronically treated patients with actively resorbing osteoclasts within lytic lesions will result in exposure of the tumor cells to high levels of bisphosphonates. Therefore, it is likely that metastatic tumor cells in patients could be exposed to high doses of clodronate for long periods of time.

This is the first time that clodronate is reported to exert a significant proliferative action on MCF-7 breast cancer cells cultured in SFM for 2 or 3 days. Previous studies examining the influence of clodronate on MCF-7 cell growth have shown either no effect, a modest inhibition of cell growth after short exposure in serum-free medium or a moderate one after high-dose exposure or prolonged treatment (more than 6 days) in serum-containing medium (Busch *et al*, 1998; Fromigue *et al*, 2000; Senaratne *et al*, 2000). The weak proapoptotic effects of clodronate that has been occasionally observed could be attributed to the accumulation of toxic analogues of ATP generated by bisphosphonate metabolism (Frith *et al*, 1997). By contrast, in our study, clodronate showed no effect on cells grown in medium containing complete serum, suggesting that serum growth factors may prevent clodronate-induced apoptosis, and that serum steroids may obscure clodronate-induced cell growth.

Importantly, the oestrogenic stimulation induced by clodronate was not cell line specific. In SFM, we indeed obtained similar results using the IBEP-2 cell line which was previously established in our laboratory from breast carcinoma (Siwek *et al*, 1998). IBEP-2 cells have recently been characterised as an oestrogen-responsive cell line closely resembling the MCF-7 cells with regard to ER expression, cell mitogenic response to oestrogenic stimulation and sensitivity to antioestrogens (Journe *et al*, 2004a). We thus observed that clodronate stimulated IBEP-2 cell proliferation, and also provoked ER downregulation and induced PgR expression (two last data not shown). By contrast, the MDA-MD-231 cells, which do not express functional ER, exhibited no oestrogenic response to the bisphosphonate in SFM. Furthermore, at the highest concentration, clodronate inhibited MDA-MB-231 cell growth, probably because of the accumulation of nonhydrolysable analogues of ATP. Consequently, it appears that the mitogenic actions of clodronate can only be detected in oestrogen-responsive breast cancer cells cultured in SFM, the sole conditions appropriate for the observation of E_2_-induced stimulation.

Clodronate belongs to the group of non-nitrogen bisphosphonates closely resembling pyrophosphate. As mentioned above, it can be metabolised to nonhydrolysable analogues of ATP affecting numerous biosynthetic, metabolic or signal transduction pathways involving the hydrolysis or synthesis of ATP, including DNA synthesis, glycolysis and protein phosphorylation (Frith *et al*, 1997). Accumulation of toxic ATP analogues could account for the weak toxicity of clodronate reported in previous studies (Fromigue *et al*, 2000; Senaratne *et al*, 2000). However, in SFM, it is likely that the weak toxicity of nonhydrolysable ATP analogues may be masked by the oestrogenic effect of clodronate, as we describe in oestrogen-sensitive cell lines. The molecular mechanisms by which clodronate stimulates MCF-7 cell growth through ER activation in SFM remain a matter of speculation. Direct interaction of clodronate with ER, leading to the activation of the receptor, is conceivable. Indeed, pyrophosphate has been reported to enhance E_2_-mediated binding of rat uterine ER to oligodeoxythymidylate cellulose (Myatt *et al*, 1985). On the other hand, because of its close structural similarity with pyrophosphate, clodronate could alter intracellular processes involving protein phosphorylation/dephosphorylation. An attractive possibility would be that clodronate interferes with the activity of phosphatases similar to alendronate or tiludronate in osteoclasts (Schmidt *et al*, 1996; Murakami *et al*, 1997), and causes subsequent accumulation of phosphorylated key proteins implicated in mitogenic signaling pathways such as ERK1/2. Supporting this view, pervanadate, a potent inhibitor of tyrosine phosphatases, was reported to activate ERK1/2 (Zhao *et al*, 1996), and etidronate, another pyrophosphate-like bisphosphonate, has been reported to produce a rapid and transient increase in the phosphorylation of ERK1/2 (Plotkin *et al*, 1999). Hence, clodronate could act as a phosphatase inhibitor promoting ERK1/2 phosphorylation, which in turn could activate ER and stimulate its binding to DNA (at ERE and/or AP-1 sites), inducing gene transcription and further cell proliferation (Kushner *et al*, 2000). Of note, several lines of evidence indicate that E_2_ can also increase ERK1/2 phosphorylation in MCF-7 cells, thereby promoting cell growth through the activation of the MAPK pathway (Improta-Brears *et al*, 1999; Driggers and Segars, 2002). Further experiments are warranted to confirm this hypothesis.

By contrast, nitrogen-containing bisphosphonates (e.g. pamidronate, ibandronate and zoledronic acid) exhibit cytotoxic effects by interfering with the mevalonate pathway, leading to inhibition of the post-translational prenylation of small GTP-binding proteins such as Ras, that are necessary for cell function and survival (Luckman *et al*, 1998; Russell and Rogers, 1999). The antitumoral activity of nitrogen-containing bisphosphonates on breast cancer cells is now largely documented and has been associated with induction of apoptosis (Fromigue *et al*, 2000; Senaratne *et al*, 2000; Jagdev *et al*, 2001). Differences between clodronate and nitrogen-containing bisphosphonate actions were also observed in steroid-free culture conditions since 10^−4^ M ibandronate strongly inhibited MCF-7 cell growth (IC_50_=10^−4^ M) and did not affect ER expression or activity (Journe *et al*, 2004b).

In conclusion, we report a previously unknown mitogenic effect of the bisphosphonate clodronate on MCF-7 breast cancer cells cultured in SFM, an environment mimicking that created by the administration of AIs *in vivo*. Even though obtained by *in vitro* studies, our observations raise the possibility of growth stimulation of ER-positive breast cancer in patients receiving AIs and clodronate, a combined therapy that will probably be used extensively in future. Our data thus suggest that caution has to be exercised before extensive combined clinical use of AIs with clodronate. Further experimental work must, however, be undertaken to establish the relevance of our *in vitro* observations.
